# No evidence of differential effects of SFA, MUFA or PUFA on post-ingestive satiety and energy intake: a randomised trial of fatty acid saturation

**DOI:** 10.1186/1475-2891-9-24

**Published:** 2010-05-24

**Authors:** Caroline M Strik, Fiona E Lithander, Anne-Thea McGill, Alastair K MacGibbon, Brian H McArdle, Sally D Poppitt

**Affiliations:** 1 Human Nutrition Unit, University of Auckland, Auckland, New Zealand; 2 Trinity College, Dublin, Ireland; 3 Human Nutrition Unit & School of Public Health, University of Auckland, Auckland, New Zealand; 4 Fonterra Research Centre, Palmerston North, New Zealand; 5 Department of Statistics, University of Auckland, Auckland, New Zealand; 6 Human Nutrition Unit, School of Biological Sciences & Department of Medicine, University of Auckland, Auckland, New Zealand

## Abstract

**Background:**

High fat diets have long been associated with weight gain and obesity, and the weak satiety response elicited in response to dietary lipids is likely to play a role. Suppression of appetite and food intake has consistently been shown to be diminished with high fat relative to either high protein or carbohydrate meals. There is however some evidence that the satiating capacity of lipids may be modulated when physicochemical properties are altered, but studies investigating the effect of lipid saturation on appetite have generated inconsistent findings. This study investigated the effects of changes in fatty acid saturation on post-ingestive satiety and energy intake.

**Methods:**

High-fat (HF) test breakfasts (2.0 MJ) containing 26 g lipid were given to 18 healthy, lean men in a 3 treatment randomised cross-over design, each treatment separated by a washout of at least 3 days. The breakfasts were high in saturated (SFA, 65% of total fat), polyunsaturated (PUFA, 76%) or monounsaturated (MUFA, 76%) fatty acids, and comprised 2 savoury muffins. Participants rated appetite sensations using visual analogue scales (VAS) to assess palatability immediately following the meals, and hunger and fullness prior to the HF breakfast and throughout the day. Energy intake was measured by covert weighing of a lunch meal which was served 3.5 h after the breakfast, and from which the participants ate *ad libitum*.

**Results:**

There was no difference in VAS ratings of pleasantness, visual appearance, smell, taste, aftertaste and overall palatability between the 3 high-fat test breakfasts. However, there was also no differential effect of the 3 treatments on ratings of hunger, fullness, satisfaction or prospective food consumption during the 3.5 h following the breakfast meal and over the full 6 h experiment. Energy and macronutrient intake at lunch also did not differ between treatments (mean, sem; SFA: 5275.9 ± 286.5 kJ; PUFA: 5227.7 ± 403.9 kJ; MUFA: 5215.6 ± 329.5 kJ; P > 0.05). The maximum difference in energy intake between treatments was less than 2%.

**Conclusions:**

There was no evidence of a difference in post-ingestion satiety between high fat meals which differed in saturation profile in this group of lean, healthy men.

**Trial Registration:**

ACTRN12610000193077

## Introduction

A high intake of dietary fat has long been implicated in the development of obesity with a positive association between a high-fat (HF), high-energy dense diet and a high body mass index (BMI) [1,2]. Whether there is a causal relationship between dietary fat and the current high levels of obesity continues to be debated however, [3-8] as does dietary fat as a primary driver of hyperphagia, or overconsumption, during weight gain. In a number of early studies dietary fat was shown to exert a weaker satiating effect than isoenergetic amounts of either carbohydrate (CHO) or protein in most [9-11], although not all [12,13] studies. Possibly factors such as palatability [14], high energy density [10,11,15-17] and relatively weak oxidative feedback of dietary lipids [15,18-20] may play a role in overconsumption of lipid-rich foods [10], the latter hypothesis having been recently revisited [21]. The inability of lipids to mount a strong postprandial satiety-related hormone response relative to the other macronutrients may also contribute [17,22,23].

There is also some evidence to suggest that the association of a HF diet with weight gain and obesity may in part depend on the saturation of the fatty acids (FA) consumed and that unsaturated, and in particular polyunsaturated FAs (PUFA), may be associated with lower adiposity [24-26]. Several studies have shown that the rate of postprandial fat oxidation, purported to act as a satiety feedback signal, is negatively related to the degree of saturation [27-33]. In early studies degree of saturation was also linked with changes in putative appetite modifiers such as CCK [34], insulin and serotonin [35]. In addition, effects on *ad libitum *energy intake (EI) have been reported. Whilst there is some evidence of PUFA having the strongest and MUFA the weakest suppression of food intake, clinical studies are inconsistent and variable. A gastrointestinal infusion study showed high-PUFA Intralipid^® ^(a fat emulsion comprising largely soybean oil) and linoleic acid (18:2) to decrease intake when compared with a no-fat saline control, whilst carbon chain length (CCL) -matched stearic acid (C18:0, SFA) and oleic acid (C18:1, MUFA) did not [36]. In a feeding study of CCL-matched fats, both high-PUFA and high-SFA meals decreased food intake when compared with high-MUFA meals [37], and a second infusion study showed lauric acid (C12:0 SFA) but not the longer chain oleic acid (C18:1 MUFA) to decrease food intake relative to a saline control [38]. Conversely, other studies have found less convincing [39,40] or no evidence of saturation affecting *ad libitum *EI or appetite ratings [41-43], or with response differing by gender [39].

This lack of consensus led to our current study which investigated whether changes in the saturation profile of a high-fat breakfast meal affected postprandial appetite sensations and food consumption at the subsequent meal using a study design which we have previously shown to be sensitive to manipulations in energy and fat content [44]. Based on prior studies we hypothesized that there may be a satiety hierarchy of PUFA > SFA > MUFA when a high fat meal balanced for both energy and total fat content is consumed.

## Participants and Methods

### Participants

Lean male participants (BMI 18-25 kg/m^2^), aged between 18-55 years were recruited into this intervention trial through poster, newspaper and electronic advertisement. Body weight and height were measured whilst fasted on 2 consecutive occasions and mean BMI recorded. Participants were from the local community including tertiary institutions. Exclusion criteria were self-reported current or previous history of overweight or obesity, current or recent history (previous 6 months) of dieting including commercial weight loss programs or weight loss surgery, eating disorders or significant restraint [45], smoking, hypertension, cardiovascular disease, diabetes mellitus (type I or II), and any significant metabolic, endocrine or gastrointestinal disease. Normal lipid profile, full blood count and fasting blood glucose levels were also ensured at screen. None of the participants were taking medications known to affect appetite or weight regulation. Written consent was obtained from each of the participants. Ethical approval for this study was obtained from the Northern Regional Ethics Committee, Auckland, New Zealand.

### Experimental design

This was a randomised, 3 treatment cross-over trial where the effects of a HF test breakfast supplemented with SFA, PUFA or MUFA were assessed through subjective visual analogue scales (VAS) and *ad libitum *EI at a single lunch meal. The primary objective of measurement of food intake at the lunch meal was not revealed to the participants prior to the study. All participants attended the Human Nutrition Unit (HNU) on three separate occasions between which they returned home for a washout period of at least 3 days where they were asked to resume their habitual diet and exercise pattern. Participants were asked to abstain from alcohol and strenuous physical activity for 24 hours prior to the study-day, and to complete a 24-hour dietary recall and physical activity questionnaires. Physical activity was estimated using a questionnaire evaluating activities according to duration and intensity using the PEPSA (Physical Exercise Programme for Sedentary Adults) points system [46] to assess whether differences in activity on the day prior to the test may have confounded measured outcomes of appetite.

Participants were asked to fast from 2000 h the previous evening, including liquids, not to exercise in the morning and to arrive at the Unit using motorized transport. At the clinic body weight and waist circumference were measured fasted, and adverse events and/or medications used between treatments recorded. Immediately upon arrival at 7 am the participants were given 200 ml water which they were required to drink in full, after which they were asked to complete baseline VAS rating their subjective feelings of hunger, fullness, satisfaction and prospective food consumption [47]. A venous cannula was also inserted for sequential collection of blood samples throughout the morning, and lipid profile, glucose and insulin concentrations were measured. The HF test breakfast was served at 0830 h and participants were asked to consume the test-meal in full but at their own pace within a 15 minute period. The exact duration of the meal was recorded. No further foods were allowed throughout the morning until an *ad lib *lunch meal was served 210 minutes later, at 1200 h, to assess energy and macronutrient intake. Lunch was served individually in a quiet dining room with minimal distractions. VAS ratings were measured throughout the morning and for 2 hours after completion of the *ad lib *lunch. All measurements of food intake were covert. Participants remained at the HNU during each study-day and were allowed to read, write or undertake other such sedentary activities but were not allowed to sleep.

### High-fat test breakfasts

The 3 breakfast treatments were HF (50 en% fat) and isoenergetic (2.0 MJ), comprising 2 savoury muffins (ingredients: flour, skimmed milk powder, eggs, tomato, ham, test lipid). The lipids were included as an integral part of the recipe and baked into the muffins. The test breakfast was served at 0830 h, co-presented with 300 ml water. The treatments differed in FA composition and were (i) high-SFA (from butter fat), (ii) high-PUFA (from safflower oil) or (iii) high-MUFA (from olive oil). The energy and macronutrient composition of the 3 test breakfasts was calculated using the dietary program FoodWorks™ (Professional Edition, Version 2.10.136, 1998-2000; Xyris Software) and are shown in Table 1.

**Table 1:** Energy and macronutrient composition of the 3 high-fat breakfast test meals

	High-SFA (Butter fat)	High-PUFA (Safflower oil)	High-MUFA (Olive oil)
Weight, as eaten (g)	151	151	145
Energy (kJ)	1974	1952	1955
Energy density (kJ/g)	13.1	12.9	13.4
Total Fat (g)	26.5	25.9	26.1
Protein (g)	15.9	15.7	15.7
CHO (g)	41.9	41.7	41.8
C12:0	0.8	0	0
C14:0	2.7	0	0
C16:0	6.8	1.8	2.9
C18:0	3.4	0.7	0.8
other	3.4	0.1	0.2
*ΣSFA*	17.1	2.6	3.8
C18:2n6	2.1	19.7	2.4
C18:3n3	0.2	0.1	0.2
*ΣPUFA*	2.2	19.8	2.5
C18:1	6.4	3.7	19.5
other	0.7	0	0.2
*ΣMUFA*	7.1	3.7	19.8
kJ from Fat (%)	50.0	49.4	49.4
kJ from Protein (%)	13.9	13.7	13.7
kJ from CHO (%)	34.3	34.2	34.2
Fat as SFA (%)	64.8	10.0	14.6
Fat as PUFA (%)	8.4	75.7	9.6
Fat as MUFA (%)	26.7	14.2	75.8

SFA, saturated fatty acids; PUFA, polyunsaturated fatty acids; MUFA, monounsaturated fatty acids; CHO, carbohydrate

### Visual Analogue Scales

Participants rated their hunger, fullness, satisfaction and prospective consumption (how much do you think you could eat now?) using VAS [47]. Subjective feelings were recorded by placing a vertical line across 100 mm scales, anchored at either end by statements; "I am not hungry at all/I am not full at all/I am completely empty/Nothing at all" on the left and "I am as hungry I have ever been/I am totally full/I cannot eat another bite/A large amount" on the right. VAS rating how thirsty, energetic and relaxed the participants felt were included as a distraction from the main outcome. VAS were completed prior to the test meal and 15, 30, 45, 60, 90, 120, 150, 180, 210, [*ad lib *lunch], 270, 330 and 390 minutes after the participant began consumption of the test breakfast. Immediately after breakfast, participants also rated the pleasantness, visual appeal, smell, taste, aftertaste and overall palatability of the breakfast using separate 100-mm VAS [9,47]. These questions were anchored on the left by each of the statements "not at all pleasant/bad visual appeal/bad smell/bad taste/strong aftertaste/bad palatability" and on the right by the statements "as pleasant as I have ever tasted/good visual appeal/good smell/good taste/no aftertaste/good palatability".

### *Ad libitum *lunch

The *ad libitum *lunch consisted of a restricted buffet-style meal with predominantly cold and one hot meal choice, along with a selection of beverages. In an attempt to avoid over-consumption the variation of meal items offered was limited. Participants were advised that they could eat as much or as little as they chose and that they were to remain in the room for a period of 45 minutes. The items presented at the *ad lib *buffet meal, along with their serving weight, energy and macronutrient content are shown in Table 2. All discrete items (eg bread) were presented as small portions (e.g. quarter slices of bread), and all items were served in excess. Covert weighing of each meal item was carried out before and after lunch to allow calculation of energy and macronutrient intake. Food items were weighed to the nearest 0.5 g (Sartorius AG, Goettingen, Germany), and energy and macronutrient content of foods consumed calculated using the dietary program FoodWorks (Professional Edition Version 2.10.136 1998-2000; Xyris Software).

**Table 2:** Energy content and macronutrient composition of foods offered to participants at the *ad libitum *lunch meal

Menu Items	Portion Size (g)	No. of typical serves	Energy (kJ)	Protein (g)	Fat (g)	CHO (g)
**Main meal items**						
Fried rice	1600	8	4621	39.0	29.8	167.4
Bread, light rye, quarter slices	168	2	1781	13.3	3.2	62.0
Bread, white, quarter slices	168	2	1814	13.6	3.2	83.8
Chicken breast, flesh, roasted, shredded	190	8	1170	48.1	9.8	0
Smoked ham slices, chopped	190	8	888	30.4	3.8	13.3
Capsicum red & green, raw	68	1	72	0.9	0.2	3.0
Tomatoes, raw	127	1	86	20.0	0.3	3.4
Spiced apple and fruit loaf, quarter slices	400	10	5620	20.0	45.2	212.4
Peach slices, tinned in fruit juice, drained	820	4	1476	4.1	0.8	77.9
**Condiments**						
Butter	250	50	6457	2.5	175.0	3.5
Mayonnaise	250	10	3675	2.0	76.8	44.5
Soy sauce	300	10	297	3.0	0	15.0
**Drinks**						
Cola drink	1500	10	2943	0	0	178.2
Orange juice	1000	5	1027	2.1	3.1	52.9
Milk, full fat (for decaffein- ated tea/coffee)	1000	20	2550	31.0	33.0	47.0
Water, bottled	1500	10	0	0	0	0

CHO, carbohydrate

### Statistical Analysis

VAS data were analysed using a repeated measures Linear Mixed Model ANOVA (SAS: PROC MIXED, SAS version 8.0, SAS Institute Inc, Cary, NC, 2001). The participant, the dietary treatment, the intervention period, and the study day were included in the procedure, as was the treatment/time interaction which addressed whether the trajectory over time during the intervention period differed between treatments (diet*time). MANOVA was also used to generate a full model for VAS incorporating all dependent variables. Energy and macronutrient intake data from the *ad lib *lunch meal was analysed using univariate ANOVA (SAS: PROC MIXED, SAS version 8.0, SAS Institute Inc, Cary, NC, 2001). Univariate ANOVA was also used to determine any differences in EI or physical activity level on the day prior to each treatment visit (Day-1). The trial was analysed on the basis of intention to treat (ITT), hence all data from all treatment visits completed were included in the analysis. Missing data was assumed missing at random and no data imputation was performed. Statistical significance was based on 95% limits (P < 0.05). Participant characteristics are presented as mean, standard deviation (mean, SD). Efficacy endpoints are presented as mean, standard error of the mean (mean, SEM).

## Results

### Participants

Eighteen (18) male participants were randomized into the study, of which 17 completed all 3 treatments. One participant completed only 2 treatments (SFA, MUFA; PUFA treatment was not completed) due to relocation overseas, hence a total of 53 of the scheduled 54 study visits were completed. Participant characteristics are shown in Table 3. They were on average young, lean men. All participants were normoglycaemic with no evidence of hyperlipidaemia or hypertension. On the day prior to intervention there was no significant difference in reported EI (mean, SEM) between treatments (SFA: 8338, 738 kJ/day; PUFA: 8259, 640 kJ/day; MUFA: 7919, 790 kJ/day; treatment, P > 0.05) or reported level of physical activity (treatment, P > 0.05). Physical activity level (mean, SEM) was assessed through hours spent on mild-moderate activity (SFA: 2.1, 0.4 h; PUFA: 2.4, 0.4 h; MUFA: 2.8, 0.6 h), minutes spent on vigorous-strenuous activity (SFA: 4.2, 2.9 m; PUFA: 8.8, 5.6 m; MUFA:10.8, 5.7 m), hours standing (SFA: 4.1, 0.7 h; PUFA: 3.4, 0.5 h; MUFA: 4.0, 0.6 h), hours sitting and watching TV/video (SFA: 4.5, 0.9 h; PUFA: 5.1, 0.8 h; MUFA: 4.2, 0.9 h), and total PEPSA points (SFA:16.9, 4.3; PUFA: 21.2, 6.5; MUFA: 28.1, 10.2). Between participants results were highly variable and there were no significant treatment effects (treatment, P > 0.05).

**Table 3:** Participant characteristics at baseline.

Baseline	Mean	SD
n	18	
Age (y)	27.8	8.0
Body weight (kg)	69.3	6.4
BMI (kg/m^2^)	22.0	1.9
Waist circumference (cm)	75.2	3.9
TC (mmol/L)	4.2	0.7
LDL-C (mmol/L)	2.2	0.5
HDL-C (mmol/L)	1.6	0.3
TAG (mmol/L)	0.9	0.4
TC:HDL-C ratio	2.7	0.5
Glucose (mmol/L)	4.5	0.3
SBP (mm Hg)	108	13
DBP (mm Hg)	64	8

All measurements made at screen visit.  Mean (SD). BMI, body mass index; TC, total cholesterol; LDL-C, low density lipoprotein cholesterol; HDL-C, high density lipoprotein cholesterol; TAG, triacylglycerol; SBP, systolic blood pressure; DBP, diastolic blood pressure

### Visual Analogue Scales

**Figure 1:**
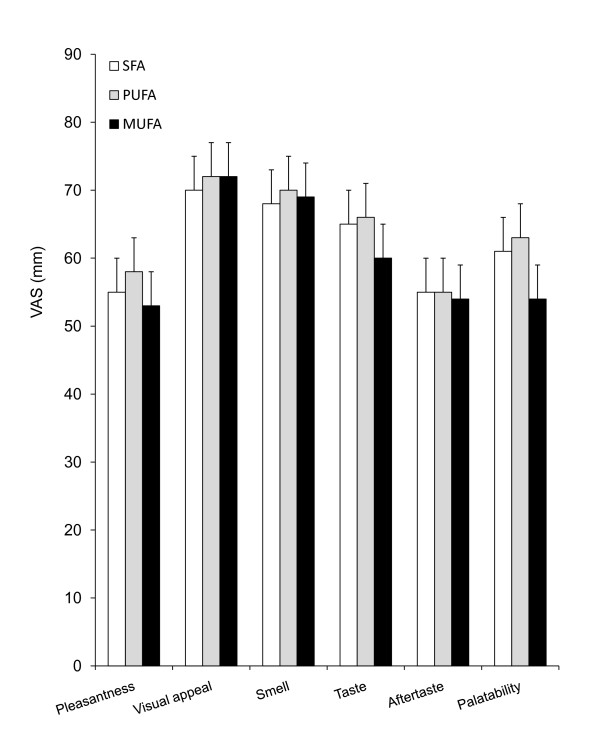
**There was no difference between mean (SEM) VAS ratings of pleasantness, visual appeal, smell, taste, aftertaste or palatability between the 3 test-breakfasts when assessed immediately after the meal (treatment, P > 0.05)**. SFA, high-saturated fatty acids (n = 18); PUFA, high-polyunsaturated fatty acids (n = 17), MUFA, high-monounsaturated fatty acids (n = 18).

**Figure 2:**
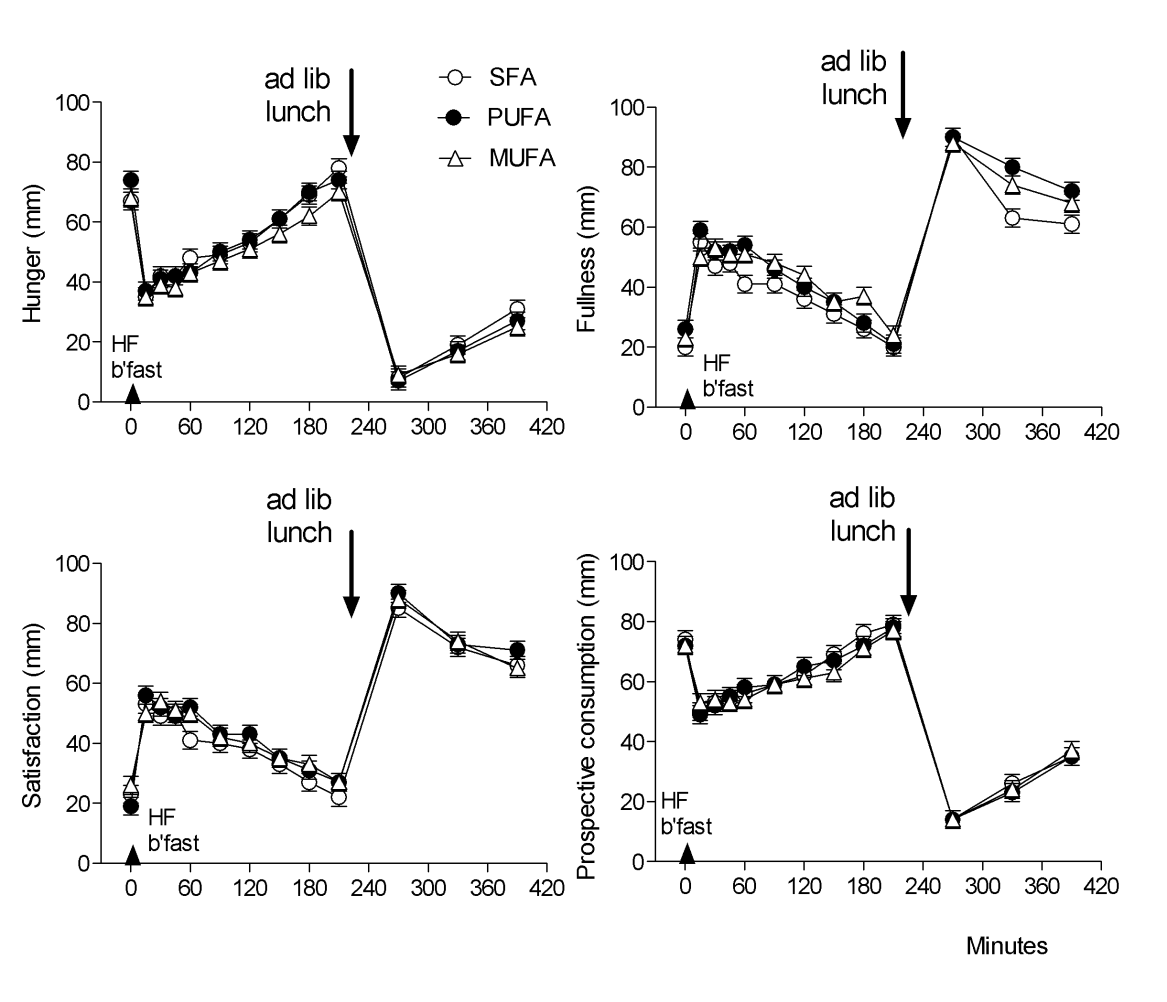
**Mean (SEM) VAS for hunger, fullness, satisfaction and prospective consumption between 0-390 minutes**. Each high lipid test breakfast was given immediately after the baseline (t = 0 mins) VAS, and the *ad libitum *lunch was served at 210 minutes. SFA, high-saturated fatty acids (n = 18); PUFA, high-polyunsaturated fatty acids (n = 17), MUFA, high-monounsaturated fatty acids (n = 18).

There was no difference in VAS ratings of pleasantness, visual appearance, smell, taste, aftertaste and overall palatability between the 3 HF test breakfasts when assessed immediately following consumption (treatment, P > 0.05, Figure 1), indicating that participants were unable to differentiate between the saturation profiles using visual and sensory cues in this blinded trial. VAS were also used to assess subjective feelings of hunger and satiety throughout the experiment. Figure 2 shows the mean ratings for hunger, fullness, satisfaction and prospective food consumption during the 6 hours following the test breakfast for each of the high-SFA, high-PUFA and high-MUFA treatments. As expected following a 2MJ meal, hunger and prospective consumption significantly decreased and fullness and satisfaction significantly increased immediately following each of the treatments, and then gradually returned to baseline by 210 mins (time, P < 0.01). Consumption of the *ad lib *lunch also induced significant changes in VAS-rated measures of appetite and satiety during the afternoon. There was however no significant difference in mean ratings of hunger, fullness, satisfaction or prospective consumption between the 3 treatments when measured over the 3.5 hours between the test breakfast and *ad lib *lunch meal (0-210 mins; all, treatment*time P > 0.05) or when measured throughout the full study day (0-390 mins; all, treatment*time P > 0.05). The use of MANOVA to combine VAS measurements for all treatments was also unable to identify a significant difference between treatments either before lunch or over the full study day (treatment*time, P > 0.05). Analysis of the sequential blood collects also showed there to be no difference between circulating levels of serum cholesterol, triacylglycerol (TAG), glucose or insulin between the SFA, MUFA and PUFA treatments (all, treatment*time P > 0.05).

### Energy and macronutrient intake at the *ad libitum *lunch

**Figure 3:**
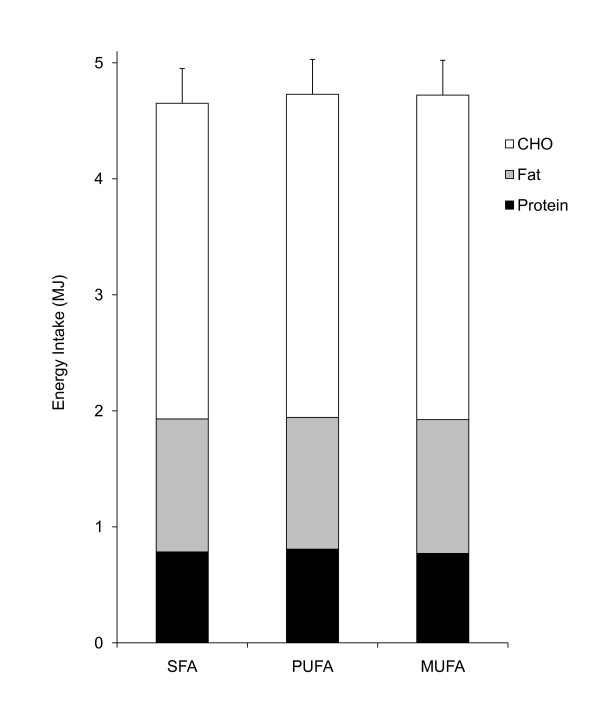
**Mean (SEM) energy intake at the *ad libitum *lunch meal, showing the macronutrient components consumed**. SFA, high-saturated fatty acids (n = 18); PUFA, high-polyunsaturated fatty acids (n = 17), MUFA, high-monounsaturated fatty acids (n = 18).

Mean total EI and energy contributed by CHO, fat and protein respectively at the *ad lib *lunch is presented for each treatment in Figure 3. There was no significant difference in total EI between lipid treatments (treatment, P > 0.05). Mean (SEM) EI at lunch was 5275.9 (286.5) kJ, 5227.7 (403.9) kJ, and 5215.6 (329.5) kJ following the SFA-, PUFA-, and MUFA-rich breakfasts respectively. The maximum difference between treatments was less than 2%. When macronutrient intake was compared between treatments there was no significant difference in CHO, fat or protein nor weight of food consumed at the lunch meal (treatment, P > 0.05).

## Discussion

There was no evidence from this study that changes made to the fatty acid saturation of a high fat breakfast altered subjective hunger ratings or energy intake at the subsequent lunch meal in a group of healthy male participants. This is consistent with a number [41-43] but not all [36-38,40] previously published studies, and there are several issues concerning study design which it is important to consider.

There are a wide range of published methods by which short-term appetite regulation has been assessed but consensus is yet to be achieved as to best practice [48]. The methodology in our current trial was based primarily on the lipid emulsion trials of Burns and colleagues [49-51] where small manipulations in lipids at a test breakfast induced significant changes in EI at a subsequent lunch meal. Arguably, the long separation between the intervention and the *ad lib *lunch (>3 hours) in these trials may make changes in eating behaviour more difficult to effect than studies where an outcome meal is given only 60 or 90 minutes following the test treatment. In our current trial however the *ad lib *energy intake was consistent with the recorded postprandial feelings of hunger and fullness which also did not differ across lipid treatments. It is possible that the varied item buffet-style lunch may have encouraged overconsumption, decreased sensitivity and masked differential effects, but again it is notable that a multi-item buffet lunch has previously been shown to be sensitive to effects of acute lipid manipulations [49-51]. We have previously shown it to be sensitive to changes in energy content at a breakfast meal [44]. Pleasantness, visual appeal, smell, taste, after-taste and palatability were all well matched between treatments in our current trial, and differences in the energy density of the test lipids were small.

There is little consensus as to whether degree of lipid saturation may play a role in regulation of body weight and adiposity through mechanisms driving changes in either intake or oxidation. Whilst epidemiological evidence of an association between lipid saturation and adiposity in studies such as the Quebec Family Study [24] suggest that saturation may influence either intake or oxidation, evidence remains scant. Friedman originally proposed the hypothesis that oxidation of macronutrients following a meal may exert a negative feedback on appetite and eating behavior, and hence control the satiating power of a given nutrient [18]. Whilst human studies have been unable confirm this mechanism [17], there are some data which do show an oxidative gradient may exist and may be driven in part by FA saturation [25,27-33] and hence the hypothesis remains of interest [21]. Interestingly, FA saturation has also been shown to impact postprandial hormone response including the peptides insulin [52] and CCK [34,36] which may drive changes in intake, although again the data remains inconclusive [53-55].

Much of the evidence for effect of saturation on EI has been derived from acute studies of intake of similar design to our current trial and also from acute GI infusion trials, several of which show increased satiety effects of PUFA. Intestinal infusion of Intralipid (54% PUFA linoleic acid, 29% MUFA oleic acid) and PUFA (linoleic acid), but not SFA (stearic acid) or MUFA (oleic acid) emulsions tended to decrease EI relative to a saline control in a study of men given a restricted lunch meal [36]. The effects were modest however with no significant differential effects between the 4 fat loaded treatments. This was supported in part by Lawton *et al *(2000) in a CCL-matched feeding study which demonstrated decreased ratings of appetite and a trend, albeit non-significant, towards lower *ad lib *EI over the rest of the day following a high-PUFA (linoleic acid) and SFA (stearic-oleic blend) lunch meal compared with a lunch rich in MUFA (oleic acid) [37]. A second study by the same authors was unable to confirm these results, although merging data from the 2 studies did support the greater satiety effects of PUFA [37]. A more recent intraduodenal infusion study of the C12:0 SFA lauric acid versus longer chain C18:1 MUFA oleic acid showed a decrease in EI following the SFA but not MUFA treatment relative to a saline control [38]. Conversely, there are also studies which find MUFA to be more satiating than PUFA [39,40]. For example, Burton-Freeman *et al *(2005) found that mean VAS scores of appetite satisfaction were greater following a high-oleic safflower oil (high-MUFA) compared with a walnut oil (high-PUFA) preload, although it is notable that this result was not substantiated by 4 other appetite-related VAS or subsequent *ad lib *EI at lunch [39]. Finally there are further trials which are consistent with our current study and which have been unable to detect a difference in ratings of appetite or EI between meals of varying saturation profile [41-43]. It is apparent that a clear effect of lipid saturation on satiety has yet to be established.

Some variability in outcome may in part be due to differences in methods. Important aspects of trials assessing postprandial changes in appetite may include number and characteristics of participants; lipid dose, composition and route of administration; inter-meal interval; and composition and variety of foods offered at the *ad lib *outcome meal. These have been summarized in Table 4. Of the trials so far conducted, all were small sample studies (8-25 participants) conducted predominantly in lean, healthy participants. Dose of lipid administered does not appear to unduly bias outcome since studies by Lawton [37] and Flint [42] both administered high doses of lipid (>50 g fat), yet only the Lawton study conducted in lean participants showed differential effects of FA saturation. The fact that delivery of lower doses, such as our current trial (26 g), are sufficient to elicit a response was demonstrated by Kamphuis and colleagues where a 25 g lipid supplement differentially altered EI [40]. Burton-Freeman [39] administered a low dose (9-13 g) lipid treatment yet observed significant effects on some VAS-assessed satiety ratings, albeit these did not result in suppression of EI at a subsequent test meal. The route of lipid administration is likely to also be important and it is notable that 2 [38,56] of the 4 [37,40] trials where a differential response was elicited delivered the lipid treatments via GI infusion hence bypassing early sensory and cognitive signaling. Whilst it is difficult to find methodological issues directly responsible for the variable outcome of the studies to date, each of the trials appears robust in its approach, it is clear that a strong case cannot be built for enhanced satiety with high-SFA, MUFA or PUFA based on current evidence.

**Table 4:** Previous studies investigating the effect of fatty acid saturation on subjective appetite ratings and *ad libitum *energy intake (measured or from diet records) at a meal, highlighting methodological differences between trials

Publication	Participants	Lipid dose	Lipid composition [% of total lipid]	Inter-meal interval	Study endpoint	Study outcome
Current study: Strik *et al*., 2010	Lean, men, n = 18	26 g, [50 en% fat]	Butter fat, high stearic-lauric blend [65% SFA]; Olive oil, high oleic acid [76% MUFA]; Safflower oil, linoleic acid [76% PUFA]	210 mins	*Ad libitum *EI from lunch meal	No effect of saturation on EI
Lawton *et al*., 2000 [37]	Lean, men, n = 10 women, n = 10	Women 58 g; Men 83 g; [55 en% fat]	Stearic-oleic blend [44% SFA, 44% MUFA]; High-oleic oil [81% MUFA]; High linoleic oil [75% PUFA]	240 mins	*Ad libitum *EI from buffet dinner + snack boxes	PUFA and SFA tended to decrease EI relative to MUFA (trend only)
French *et al*.,2000 [36]	Lean, men, n = 10	Duodenal infusion; 20 g lipid emulsion [100 en% fat], at rate of 1 mL/min over 100 mins	High stearic/oleic [40% SFA; 44% MUFA]; High oleic acid [75% MUFA]; High linoleic acid [74% PUFA]; Intralipid [16% SFA, 29% MUFA, 54% PUFA]; saline	90 mins	*Ad libitum *EI from lunch meal	PUFA decreased EI relative to SFA and MUFA (trend only); signif lower than saline control
Kamphuis *et al*., 2001 [40]	Overweight, men, n = 8; women, n = 8	20 ml (~20 g) lunch; 25 ml (~25 g) dinner added to usual diet [~36 en % fat]	High-linoleic oil [67% LA, PUFA]; High-γ-linolenic [20% GLA, PUFA]; High-oleic oil [80% MUFA]	Test lipids given as part of *ad lib *meals	*24-h ad libitum *EI: restricted choice; lunch, dinner, snacks	MUFA decreased EI relative to PUFA at test dinner; no difference over 24 h
Alfenas *et al*., 2003 [43]	Lean, men, n = 9; women, n = 11	30-40 g; [54-59 en % fat]	Butter fat [66% SFA]; Peanut oil [49% MUFA]; Canola oil [62% MUFA]; fat free	>120 mins	Diet records of EI during free feeding over 24-h [no outcome meal]	No effect of saturation on EI
MacIntosh *et al*., 2003 [41]	Lean, men, n = 10	30 g; [55 en% fat]	Butter fat [69% SFA]; Sunola oil [80% MUFA]; Sunflower oil [64% PUFA];	120 mins	*Ad libitum *EI from lunch meal + diet; diet records over rest of day	No effect of saturation on EI
Flint *et al*., 2003 [42]	Overweight men, n = 19	63-87 g; [60 en% fat]	High-oleic sunflower oil [83% MUFA]; Hydrogenated rapeseed oil [54% trans; 31% SFA]; Grape- seed oil [70% PUFA]	300 mins	*Ad libitum *EI from lunch meal	No effect of saturation on EI

Burton-Freeman *et al*., 2005 [39]	Lean, men, n = 12; women, n = 13	Men 13 g; Women 9 g; [39 en% fat]	High-oleic safflower oil [72% MUFA]; Walnut oil [66% PUFA];Ground walnuts [66% PUFA]; low fat [1.4 g fat]	45 mins	*Ad libitum *EI from lunch meal	No effect of saturation on EI
Feltrin *et al*. 2008 [38]	Lean, men, n = 13	Duodenal infusion; ~3 g lipid emulsion [100en% fat], at rate of 4 mL/min over 60 mins	Lauric acid [100% SFA]; Oleic acid [100% MUFA]; saline	60 mins	*Ad libitum *EI from lunch meal	SFA decreased EI relative to MUFA and saline control

## Conclusions

In this study we were unable to show differential changes in postprandial feelings of hunger or fullness, or changes in energy intake at a lunch meal when alterations were made to the fatty acid saturation of a high-fat breakfast. Lipid dose, the interval between test-breakfast and *ad libitum *outcome lunch, and the composition of the *ad lib *meal have all been identified as areas which may bias outcome. In this study we administered a 26 g lipid test meal which was well matched for sensory outcomes between treatments, 3.5 hours prior to a free choice lunch meal. Whether a higher dose lipid product administered as a preload rather than test meal, i.e. closer to the lunch meal, would elicit an appetite response is unknown. Certainly there is little consensus on effects of dietary lipid composition on appetite control from previous studies. Whether appetite may be altered by longer term and sustained changes in dietary fatty acid composition also remains to be demonstrated.

## List of abbreviations used

ANOVA: analysis of variance; BMI: body mass index; CCL: carbon chain length; CCK: cholecystokinin; CHO: carbohydrate; EI: energy intake; FA: fatty acid; HF: high fat; HNU: human nutrition unit; MANOVA: multivariate analysis of variance; MUFA: monounsaturated fatty acid; PEPSA: Physical Exercise Programme for Sedentary Adults; PUFA: polyunsaturated fatty acid; SD: standard deviation; SEM: standard error of the mean; SFA: saturated fatty acid; VAS: visual analogue score.

## Declaration of Competing interests

SDP, ATM, FEL, CMS, AKM received salary support and funding for this trial from LactoPharma, New Zealand. Other authors declare that they have no competing interests.

## Authors' contributions

CM Strik was responsible for patient recruitment, screening and daily management of the trial as well as data entry and contribution to manuscript preparation; FE Lithander participated in patient recruitment, screening and oversaw trial management; AT McGill was the study physician and contributed to protocol design and regulatory procedures; AKH MacGibbon contributed to the design of the trial and provision of dietary treatments; BH McArdle was responsible for all statistical analyses; SD Poppitt was the senior author, and responsible for protocol design, data interpretation, trial supervision and regulatory procedures. All authors read and approved the final manuscript.
